# A High-Fat Diet Differentially Affects the Gut Metabolism and Blood Lipids of Rats Depending on the Type of Dietary Fat and Carbohydrate

**DOI:** 10.3390/nu6020616

**Published:** 2014-02-03

**Authors:** Adam Jurgoński, Jerzy Juśkiewicz, Zenon Zduńczyk

**Affiliations:** Division of Food Science, Institute of Animal Reproduction and Food Research, Polish Academy of Sciences, 10 Tuwima street, Olsztyn 10-748, Poland; E-Mails: j.juskiewicz@pan.olsztyn.pl (J.J.); z.zdunczyk@pan.olsztyn.pl (Z.Z.)

**Keywords:** butyrate, corn starch, caecum, fructose, lard, soybean oil, cholesterol, triglycerides

## Abstract

The aim of this model study was to investigate how selected gut functions and serum lipid profile in rats on high-fat diets differed according to the type of fat (saturated *vs.* unsaturated) and carbohydrate (simple *vs.* complex). The experiment was conducted using 32 male Wistar rats distributed into 4 groups of 8 animals each. For 4 weeks, the animals were fed group-specific diets that were either rich in lard or soybean oil (16% of the diet) as the source of saturated or unsaturated fatty acids, respectively; further, each lard- and soybean oil-rich diet contained either fructose or corn starch (45.3% of the diet) as the source of simple or complex carbohydrates, respectively. Both dietary factors contributed to changes in the caecal short-chain fatty acid concentrations, especially to the butyrate concentration, which was higher in rats fed lard- and corn starch-rich diets compared to soybean oil- and fructose-rich diets, respectively. The lowest butyrate concentration was observed in rats fed the soybean oil- and fructose-rich diet. On the other hand, the lard- and fructose-rich diet *vs.* the other dietary combinations significantly increased serum total cholesterol concentration, to more than two times serum triglyceride concentration and to more than five times the atherogenic index. In conclusion, a high-fat diet rich in fructose can unfavorably affect gut metabolism when unsaturated fats are predominant in the diet or the blood lipids when a diet is rich in saturated fats.

## 1. Introduction

A dietary pattern characterized by high consumption of energy-dense foods is thought to be partly responsible for the development of obesity and cardiovascular disease (CVD). Due to its high energy density, fat has been recognized as the most important macronutrient to affect the development of the aforementioned chronic diseases. It is generally accepted that an overabundance of saturated fatty acids elevates blood cholesterol concentration, including the LDL fraction, and therefore constitutes an important CVD risk factors. On the other hand, unsaturated fatty acids are considered as to be rather favorable for cardiovascular health [1,2]. Animal models for obesity and other metabolic disorders related to CVD are mostly based on different high-fat diets that can significantly induce not only the weight gain, body fat accumulation and dyslipidaemia of the animals, but also their insulin and leptin resistance, for example. However, the extent of the induced changes depends on many factors, such as the quantity and source of fat used in the diet and the length of experimental feeding [3]. Furthermore, a high-fat diet seems to be strongly involved in the phylogenic changes of the composition of the gut microbiota in obese individuals, probably due to the overflow of dietary fat to the distal intestine [4,5].

Although the available evidence proves that replacing saturated fatty acids with unsaturated fatty acids can improve the blood lipid profile, their replacement with carbohydrates may have only a barely favorable, or even an adverse, effect, depending on such properties as the refining, complexity and glycaemic index of carbohydrates [2,6]. Fructose is an example of a simple carbohydrate commonly found in a daily diet as an ingredient of sucrose and high-fructose corn syrup. In laboratory rodents, a high dietary intake of fructose leads to a series of metabolic disturbances, such as the impairment of the blood lipid profile, the notable elevation of the triglyceride concentration, and the induction of insulin resistance and hypertension [7]. Although such evidence is less clear in humans, epidemiological studies have also suggested a link between the increased consumption of fructose and the occurrence of metabolic and cardiovascular disorders [7]. Moreover, compared to glucose, fructose absorption seems to be quantitatively limited, as it can only partly pass through the distal part of the gut. It has also been supposed that fructose may cause or aggravate symptoms in patients with functional gastrointestinal disorders, especially if ingested without glucose whose presence stimulate absorption of fructose [7,8].

Since the excessive consumption of different types of fatty acids and carbohydrates can affect gut physiology and lipid metabolism, there is the possibility of biological interactions among these dietary factors. Therefore, the aim of this model study was to investigate the effects of high-fat diets differing according to the types of fats (saturated *vs.* unsaturated) and carbohydrates (simple *vs.* complex) on selected gut functions and blood lipid profiles of rats.

## 2. Experimental Section

### 2.1. Animals and Diets

The experiment was conducted using 32 male adult Wistar rats (average body weight 376 ± 34.4 g) randomly distributed into 4 groups of 8 animals each. For 4 weeks, the animals were fed semi-purified casein diets that were rich in fat (20% of the diets). Details about the proportional composition of each group-specific diet is shown in Table 1. In group LS, the diet consisted of a high amount of lard as an abundant source of saturated fatty acids and a low amount of soybean oil (16% and 4% of the diet, respectively), as well as had a high content of corn starch representing complex carbohydrates and a low content of fructose (45.3% and 7% of the diet, respectively). Group LF received the same proportions of lard and soybean oil as group LS, while the proportions of corn starch and fructose representing simple carbohydrates were switched (7% and 45.3% of the diet, respectively). In group OS, rats were fed a diet high in soybean oil as a source of unsaturated fatty acids and low in lard (16% and 4% of the diet, respectively) while receiving a high content of corn starch and a low content of fructose (45.3% and 7% of the diet, respectively). Finally, the diet fed to rats from group OF also contained 16% of soybean oil and 4% of lard, but the dietary proportions of corn starch and fructose were 7% and 45.3%, respectively, as received by group LF. A vitamin and mineral mix was prepared in accordance with the recommendations established for the American Institute of Nutrition-93M diet. The animals were maintained individually in glass cages under a 12-h light/dark cycle, a controlled temperature of 21–22 °C, and intensive room ventilation (15 times per hour), and they had free access to water and their respective diets. The animal protocol used in this study was approved by the Institutional Animal Care and Use Committee in Olsztyn, Poland.

**Table 1:** Composition of the diets fed to the rats.

Ingredient (g/100 g)	Group			
	LS	LF	OS	OF
Casein	19.8	19.8	19.8	19.8
DL-methionine	0.2	0.2	0.2	0.2
Corn starch ^1^	45.3	7	45.3	7
Fructose ^2^	7	45.3	7	45.3
α-Cellulose ^3^	3	3	3	3
Lard ^4^	16	16	4	4
Soybean oil ^5^	4	4	16	16
Mineral mix ^6^	3.5	3.5	3.5	3.5
Vitamin mix ^6^	1	1	1	1
Choline chloride	0.2	0.2	0.2	0.2
*Metabolisable energy (kcal/g)*	*4.76*	*4.76*	*4.76*	*4.76*

^1^ AVEBE, Veendam, The Netherlands; ^2^ BIOFAN, Chorzów, Poland; ^3^ Sigma-Aldrich; ^4^ Animex Foods Ltd., Ostróda, Poland; ^5^ ZT Kruszwica SA, Kruszwica, Poland; ^6^ Recommended for AIN-93M diet [9].

### 2.2. Sample Collection and Analysis

Upon termination of the experiment, rats were weighed and anesthetized with sodium pentobarbital (50 mg/kg body weight). After laparotomy, blood samples were collected from the caudal vein, and the gut segments (small intestine and caecum) and internal organs (liver, heart and kidneys) were removed and weighed. 

Blood was allowed to clot for two hours at room temperature before centrifugation for 15 min at 380× *g*, and then the obtained serum was stored at −20 °C. The serum concentrations of triglycerides, the total cholesterol and its HDL fraction, and the activities of aspartate transaminase and alanine transaminase were estimated using reagents from Alpha Diagnostics Ltd. (Warsaw, Poland). On the basis of the serum lipid profile, the atherogenic index was calculated as previously described, using the following formula: log(triglycerides (mmol/L)/HDL-cholesterol (mmol/L)) [10]. The serum tumor necrosis factor-alpha (TNF-α) concentration was determined using an enzyme immunoassay kit (R&D Systems, Inc., Minneapolis, MA, USA). 

Mucosa from the second quarter of the small intestine was collected by scraping with glass slides on an iced glass plate. After homogenization with four parts of cold physiological saline (*v*/*w*) and centrifugation for 10 min (9503× *g*, 4 °C), the obtained supernatant was stored at −20 °C until analysis. The mucosal disaccharidase activity (sucrase, maltase and lactase) was assayed using a procedure adopted from the method of Dahlqvist [11]. An aliquot of mucosal homogenate (0.1 mL) was incubated at 37 °C with 0.1 mL of a substrate solution (0.056 mol/L sucrose or maltose in 0.2 mol/L phosphate buffer, pH 7.0). After 30 min of incubation, 0.8 mL of cold distilled water was added, and the enzymatic reaction was finally interrupted by the immersion of the test tube in boiling water for 2 min. Additionally, a blank with the same composition was prepared and immersed in boiling water without prior incubation at 37 °C. The released glucose was determined using a glucose oxidase reagent (Alpha Diagnostic Ltd., Warsaw, Poland). The disaccharidase activity was expressed as µmol of glucose liberated from the respective disaccharide per min per g of protein. The mucosal protein content was estimated using the Bradford method with bovine serum albumin as the standard. The pH of the intestinal digesta was measured using a microelectrode and a pH/ION meter (model 301; Hanna Instruments, Vila do Conde, Portugal). Fresh caecal digesta was used for the determination of ammonia content, which was extracted and trapped in a solution of boric acid, which was then determined by direct titration with sulfuric acid [12]. The concentration of short-chain fatty acids (SCFA) was measured using gas chromatography. A known amount of fresh caecal digesta was mixed with 0.2 mL of formic acid and stored at −80 °C. Afterwards, the sample was diluted with deionized water, centrifuged at 10,000 *g* for 5 min, and filtered through a 0.45 µm membrane, and then the supernatant was decanted for injection into a gas chromatograph (Shimadzu GC-14A, Shimadzu Co., Kyoto, Japan, equipped with a glass column, 2.5 m × 2.6 mm, containing 10% SP-1200/1% H3PO4 on 80/100 Chromosorb W AW; column temperature 110 °C; flame ionization detector temperature 180 °C; injector temperature 195 °C).

### 2.3. Statistical Analysis

The data were analyzed using the STATISTICA 8.0 software package (Statsoft Co., Krakow, Poland). A two-way analysis of variance (ANOVA) was applied to assess the effects of fats and carbohydrates and the interactions between these dietary factors (Fats × Carb.). If the analysis revealed a significant interaction or that both dietary factors had a significant influence (*p* ≤ 0.05), the differences among the individual groups were then analyzed with Duncan’s multiple range *post hoc* test (*p* ≤ 0.05). 

## 3. Results

Although dietary lard *vs.* soybean oil increased (in g/day: 16.7 ± 0.32 *vs.* 15.6 ± 0.33, *p* < 0.05), whereas dietary fructose *vs.* corn starch tended to increase the dietary intake of rats during the 4 weeks of feeding (in g/day: 16.6 ± 1.42 *vs.* 15.7 ± 0.36, *p* = 0.08), the body weight gains at the end of experiment were comparable among all of the tested groups (48.8 ± 2.80 g/4 weeks on average). Furthermore, the inflammatory markers assayed in the serum (alanine and aspartate transaminases, TNF-α) did not differ among the groups (data not shown).

Compared to corn starch-rich diets, fructose-rich diets increased the small intestine relative mass, measured together with contents, the pH value of ileal digesta, and the sucrase activity in the small intestinal mucosa (*p* < 0.05, Table 2). Within the caecum, an interaction between dietary fats and carbohydrates was noted when both the relative tissue and digesta masses were considered (*p* < 0.05). The highest caecal tissue mass was observed in group OF and it significantly decreased in groups LF and OS. The caecal digesta mass was also highest in group OF and significantly lower in group LF (*p* ≤ 0.05, Table 2). 

**Table 2:** Markers of the gut function of rats fed high-fat diets differing according to the types of fats and carbohydrates.

			Group ^1^				ANOVA (*p* value)		
			LS	LF	OS	OF	Fats	Carb.	Fats × Carb.
**Small intestine**									
	Mass with content ^2^		1.84 ± 0.155	1.94 ± 0.172	1.84 ± 0.169	2.01 ± 0.212	NS	<0.05	NS
	pH of ileal digesta		6.83 ± 0.611	7.02 ± 0.300	6.77 ± 0.360	7.27 ± 0.396	NS	<0.05	NS
	Mucosal enzyme activity ^3^								
		Sucrase	3.69 ± 0.568	4.46 ± 0.916	3.64 ± 0.922	4.33 ± 0.827	NS	<0.05	NS
		Maltase	22.6 ± 3.61	23.6 ± 5.71	24.6 ± 2.84	23.5 ± 5.10	NS	NS	NS
**Cecum**									
	Tissue								
		Mass ^2^	0.257 ± 0.047 ^a,b^	0.230 ± 0.033 ^b^	0.228 ± 0.015 ^b^	0.293 ± 0.095 ^a^	NS	NS	<0.05
		TBARS (nmol/g) ^4^	28.0 ± 7.47	28.4 ± 5.55	38.6 ± 18.23	33.3 ± 19.39	NS	NS	NS
	Digesta								
		Mass ^2^	0.608 ± 0.150 ^a,b^	0.443 ± 0.115 ^b^	0.529 ± 0.137 ^a,b^	0.675 ± 0.268 ^a^	NS	NS	<0.05
		pH	7.12 ± 0.203	7.20 ± 0.188	7.13 ± 0.119	7.24 ± 0.185	NS	NS	NS
		NH_3_ (mg/g)	0.328 ± 0.478	0.321 ± 0.444	0.347 ± 0.437	0.311 ± 0.340	NS	NS	NS

The results are expressed as means ± SEM (*n* = 8/group); Values not sharing the same superscript letters within a row are significantly different at *p* ≤ 0.05; ^1^ LS, lard- and corn starch-rich diet; LF, lard- and fructose-rich diet; OS, soybean oil- and corn starch-rich diet; OF, soybean oil- and fructose-rich diet; ^2^ g/100 g body weight; ^3^ µmol/min/g protein; ^4^ Thiobarbituric acid-reactive substances.

Both the dietary fats and carbohydrates contributed to changes in the total SCFA concentration in the caecal digesta of rats (*p* < 0.05 and *p* < 0.001, respectively). The highest total SCFA concentration was in group LS, while group OS had a significantly lower concentration (*p* ≤ 0.05). Similarly, the acetate concentration in the caecal digesta was influenced both by dietary fats and carbohydrates (*p* < 0.05 and *p* < 0.001, respectively) with a similar span of differences among particular groups (*p* ≤ 0.05). The type of dietary carbohydrate had significant influence on the propionate and isobutyrate concentrations in the caecal digesta (*p* < 0.001 and *p* < 0.05, respectively); however, both dietary factors had an interactive effect on their concentrations (*p* < 0.05). The highest propionate concentration was observed in the LS and OS group, whereas significantly lower concentration was found in the OF group. The lowest isobutyrate concentration was in group OF and it was significantly higher in group OS (*p* ≤ 0.05). Furthermore, the propionate profiles increased in groups fed soybean oil-rich diets *vs.* fat-rich diets (*p* < 0.05, Table 3). Both the type of dietary fat and the type of carbohydrate influenced the butyrate (*p* < 0.01 and *p* < 0.001, respectively) and valerate (*p* < 0.05 and *p* < 0.01, respectively) concentrations in the caecal digesta. The highest butyrate concentration was in group LS and it was significantly lower in the LF group, whereas the lowest butyrate concentration was in group OF (*p* ≤ 0.05). The valerate concentration was also significantly decreased in group OF and significantly increased in the LS and OS group (*p* ≤ 0.05).

**Table 3:** Short-chain fatty acid (SCFA) content in the caecal digesta of rats fed high-fat diets differing according to the types of fats and carbohydrates.

SCFA		Group ^1^				ANOVA (*p* value)		
		LS	LF	OS	OF	Fats	Carb.	Fats × Carb.
**Concentration (µmol/g)**								
	Acetate	51.2 ± 6.84 ^a^	46.3 ± 6.67 ^a,b^	49.5 ± 2.74 ^a,b^	37.3 ± 8.61 ^b^	<0.05	<0.001	NS
	Propionate	13.3 ± 2.36 ^a^	12.9 ± 2.35 ^a,b^	15.0 ± 0.77 ^a^	11.0 ± 2.42 ^b^	NS	<0.001	<0.05
	Isobutyrate	1.29 ± 0.144 ^a,b^	1.30 ± 0.133 ^a,b^	1.38 ± 0.090 ^a^	1.11 ± 0.272 ^b^	NS	<0.05	<0.05
	Butyrate	7.19 ± 0.951 ^a^	5.81 ± 0.722 ^b^	6.30 ± 0.912 ^a,b^	4.66 ± 1.238 ^c^	<0.01	<0.001	NS
	Isovalerate	0.990 ± 0.107	0.909 ± 0.181	0.937 ± 0.157	0.828 ± 0.179	NS	NS	NS
	Valerate	1.31 ± 0.105 ^a^	1.17 ± 0.118 ^a,b^	1.21 ± 0.094 ^a^	1.02 ± 0.256 ^b^	<0.05	<0.01	NS
	Total	75.4 ± 7.58 ^a^	68.4 ± 8.51 ^a,b^	74.4 ± 3.13 ^a,b^	56.0 ± 12.44 ^b^	<0.05	<0.001	NS
**Profile (%)**								
	Acetate	68	68	67	67	NS	NS	NS
	Propionate	18	19	20	20	<0.05	NS	NS
	Butyrate	10	9	8	8	NS	NS	NS

The results are expressed as means ± SEM (*n* = 8/group); Values not sharing the same superscript letters within a row are significantly different at *p* ≤ 0.05; ^1^ LS, lard- and corn starch-rich diet; LF, lard- and fructose-rich diet; OS, soybean oil- and corn starch-rich diet; OF, soybean oil- and fructose-rich diet.

The serum lipid profiles of rats fed high-fat diets differing according to the types of fats and carbohydrates are shown in Table 4. The triglyceride concentration was strongly affected both by the fat and carbohydrate type (*p* < 0.001). Considering the triglyceride concentration, an interaction between dietary fats and carbohydrates was also noted (*p* < 0.001); its level in group LF was more than two times greater than the other groups (*p* ≤ 0.05). The total cholesterol concentration and the HDL-cholesterol concentration were only affected by the fat type (*p* < 0.01); however, both dietary factors had an interactive effect on their concentrations (Fats × Carb., *p* < 0.05). The total cholesterol concentration was significantly elevated in group LF compared to the other groups; the HDL-cholesterol concentration was highest in group LF and significantly lower in group OF (*p* ≤ 0.05). On the other hand, the concentration of non-HDL cholesterol was only increased after the consumption of lard-rich diets (*p* < 0.05). On the basis of the serum lipid profile, the atherogenic index was calculated and found to be strongly influenced by both dietary factors and their interaction (*p* < 0.001). Its value was more than five times greater in group LF than in any of the other groups (*p* ≤ 0.05, Table 4). 

**Table 4:** Serum lipid profile and atherogenic index in rats fed high-fat diets differing according to the type of fats and carbohydrates.

Serum indices	Group ^1^				ANOVA (*p* value)		
	LS	LF	OS	OF	Fats	Carb.	Fats × Carb.
Triglycerides ^2^	1.72 ± 0.303 ^b^	4.42 ± 1.760 ^a^	1.44 ± 0.209 ^b^	1.42 ± 0.231 ^b^	<0.001	<0.001	<0.001
Total cholesterol ^2^	2.02 ± 0.364 ^b^	2.36 ± 0.318 ^a^	1.96 ± 0.267 ^b^	1.86 ± 0.207 ^b^	<0.05	NS	<0.05
HDL cholesterol ^2^	1.41 ± 0.275 ^a,b^	1.57 ± 0.178 ^a^	1.41 ± 0.142 ^a,b^	1.28 ± 0.179 ^b^	<0.05	NS	<0.05
Non-HDL chol. ^3^	0.608 ± 0.135	0.791 ± 0.260	0.547 ± 0.181	0.583 ± 0.103	<0.05	NS	NS
Atherogenic index ^4^	0.084 ± 0.135 ^b^	0.427 ± 0.161 ^a^	0.008 ± 0.045 ^b^	0.044 ± 0.082 ^b^	<0.001	<0.001	<0.001

The results are expressed as means ± SEM (*n* = 8/group). Values not sharing the same superscript letters within a row are significantly different at *p* ≤ 0.05. ^1^ LS, lard- and corn starch-rich diet; LF, lard- and fructose-rich diet; OS, soybean oil- and corn starch-rich diet; OF, soybean oil- and fructose-rich diet; ^2^ mmol/L; ^3^ Total cholesterol—HDL cholesterol. ^4^ log(triglycerides/HDL cholesterol).

## 4. Discussion

Effects of different fatty acids and carbohydrates on blood lipid profile and obesity and CVD development have been the subject of extensive studies in animals and humans since 1970s [13,14]. The excessive ingestion of fat still seems to be a problem in economically-developed countries together with the constantly increasing consumption of simple sugars, especially fructose [15,16]. Dietary fructose has been suggested as one of the most important lipogenic factors that can contribute to the development of metabolic syndrome [16]. For the most part, animal studies concerning metabolic effects of high-fat diets and a high-fructose diet have been performed separately [3,16]. The present study adds new knowledge in showing to what extent a high dietary fat-related metabolic changes depends on dietary fructose, as well how is the nature of high-fat induced disturbances when different dietary sources of fat and carbohydrates are present in the diet.

Studies in human subjects and laboratory animals have shown that saturated fatty acids are more obesogenic than polyunsaturated fatty acids, and several mechanisms have been proposed to explain this phenomenon, i.e., reduced resting metabolic rate, diet-induced thermogenesis, decreased usage for energy, and the resultant increased storage in adipose tissue, lowering the satiating effect of the secretion of gastrointestinal hormones [3]. In the present study, the saturation of fatty acids seems to had some effect on satiety because the dietary intake was higher in rats fed lard-rich diets than soybean oil-rich diets, both of which were isoenergetic. Lard is an abundant source of saturated fatty acids and a poor source of polyunsaturated fatty acids (39.2% and 11.2% in total, respectively) compared to soybean oil, which is especially rich in polyunsaturated fatty acids (57.7% in total), whereas saturated fatty acids constitute only 15.7% of this oil [17]. Considering the entire period of the study, rats fed lard-rich diets consumed on average 30.8 g diet more than their counterparts fed soybean oil-rich diets. However, all animals had similar body weights at the end of the experiment. The difference in dietary intake, although significant from the statistical point of view, does not seem to be crucial for the interpretation of results, but it should be taken into account in some cases. 

In the present study, increased sucrase activity was noted after the consumption of fructose-rich diets; this is in accordance with the findings of Kishi *et al.*, who observed that fructose was capable to directly increase the activity and gene expression of sucrase [18]. The increased small intestinal mass and pH value of ileal digesta after the consumption of fructose-rich diets were also observed in the present and previous studies [19]. These results support the possibilities of fructose malabsorption and fructose-induced disturbances of ileal microbiota suggested in the literature [20]. 

Changes in the composition of the gut microbiota leading to the increased ratio of *Firmicutes* to *Bacteroidetes* phylum are recognized as a typical obesity profile, which can result from the excess of dietary fat and its overflow to the distal intestine [4,5]. However, it is supposed that the type of dietary fat can also be an important factor affecting the gut microbiota. The study on mice by de Wit *et al.* showed that unlike diets rich in mono- and polyunsaturated fatty acids (olive oil and safflower oil, respectively), a high-fat diet containing palm oil, that is rich in saturated fatty acids, can reduce microbial diversity and increase the ratio of *Firmicutes* to *Bacteroidetes* phylum [5]. On the other hand, Murphy *et al.* proved that the increase in *Firmicutes* to *Bacteroidetes* ratio is connected with simultaneous decrease in SCFA concentrations; both faecal acetate and caecal propionate levels in mice were significantly lowered after 8 weeks of feeding with a high-fat diet [21]. It confirms that changes occurring in the gut microbiota under the influence of dietary fat can strictly affect its metabolic activity. In the present study, both dietary fat and carbohydrates contributed to changes in the total and some particular SCFA concentrations, which was the lowest in rats fed the soybean oil- and fructose-rich diet. Interestingly, the caecal digesta pH remained on comparable levels among all dietary groups. It may suggests that the observed differences in SCFA concentration were too little to exceed the overall buffering capacity of the digesta, which is known to depend on many components including SCFA, ammonia, calcium, lactic acid [22]. It is noteworthy, however, that SCFA, mainly acetate, propionate and butyrate, are considered as indirect nutrients for the host that have a role in the regulation of energy metabolism and many other metabolic features [4]. In the present study, the acetic acid concentration was also the lowest in rats fed the soybean oil- and fructose-rich diet. This finding could be considered a favorable change because it is believed that, after absorption, acetate stimulates cholesterol synthesis in the liver; on the other hand, a simultaneous decrease in the propionate concentration in the OF group was also observed, and this metabolite may inhibit hepatic cholesterol and fatty acid synthesis [23,24]. However, the propionate profile was significantly higher in both soybean oil-fed groups. Furthermore, the considerable drop of the butyrate concentration noted in the OF group should be considered an unfavorable change because butyrate is the preferred energetic substrate for colonocytes and promotes a normal phenotype for these cells [23]. One of dietary components that can raise butyrate and other SCFA production as well protect against colonic DNA damage is resistant starch [25]. In this study, the amount of resistant starch could be on a decidedly lower level in the fructose-rich diets than the starch-based diets, which partly explains the observed differences in SCFA concentrations. However, it remains open why the decrease of butyrate concentration was the most significant in rats fed the soybean oil- and fructose-rich diet. Perhaps it was a consequence of increased digesta hydration, and the elevated caecal digesta mass of this group supports this explanation.

In this study, the combination of dietary lard and fructose had unfavourable effects on the serum lipid profile of rats, especially on the triglyceride concentration which was more than two times higher compared to rats fed the other dietary combinations. Hyperlipidaemia as a consequence of increased hepatic fatty acid synthesis is a well-known disorder observed in rats fed a high-fructose diet [26]. Interestingly, there are suggestions in the literature that fructose transport can be modulated by dietary fat and a diet high in saturated fatty acids, but not in polyunsaturated fatty acids, to be able to enhance its intestinal absorption [27]. Therefore, it is probable that dietary lard elevated fructose influx into the liver of rats fed the lard- and fructose-rich diet, thereby increasing fructose-induced hepatic lipogenesis. However, the observed increase in dietary intake in group LF compared to both soybean oil-fed groups might have also played a role in the stimulation of hepatic lipogenesis. On the other hand, the hyperlipidemic effect of fructose did not occur in rats fed the soybean oil- and fructose-rich diet, whose triglyceride and total cholesterol concentrations were similar to those of the rats fed the starch-based diets. Recently, Hashimoto *et al.* have reported that a high-fat diet rich in soybean oil, as opposed to lard, promotes hepatic lipid accumulation by suppressed lipolysis in the peroxisomes and normal levels of VLDL secretion [28]. The authors have indicated on linoleic acid present in soybean oil as the main causative agent. Those results may partly explain why the lipogenic effect of fructose was not observed in the serum of rats fed the soybean oil-rich diet. However, the observed decrease in caloric intake in group OF *vs.* both lard-fed groups might have also reduced the effect to some extent. Furthermore, the dietary lard increased the non-HDL cholesterol concentration in which the LDL fraction, a well-known CVD risk factor, was also included. This agrees with the literature where saturated fat is recognised as increasing LDL chlesterolaemia [1,2]. Another important marker for cardiovascular health is the atherogenic index of plasma, whose values are inversely correlated with those of the lipoprotein particle size, thereby predicting atherogenicity in humans [10]. In the present study, the atherogenic index calculated on the basis of the serum lipid profile was more than five times greater as a result of the consumption of the lard- and fructose-rich diet compared to the other diets.

## 5. Conclusions

A combination of saturated fatty acid-rich lard and simple carbohydrate fructose unfavorably affected blood lipids, causing hypertriglyceridaemia, hypercholesterolaemia and considerably increasing the atherogenic index. Such disorders were not present when unsaturated fatty acid-rich soybean oil was added to the fructose diet instead of lard; however, some disturbances in bacterial metabolite formation in the caecal digesta were observed, in particular a decreased butyrate concentration. The saturation of fatty acids seems to have had some effect on satiety because the dietary intake was higher in rats fed lard-rich diets than soybean oil-rich diets. The aforementioned results suggest that a high-fat diet rich in fructose can unfavorably affect gut metabolism when unsaturated fats are predominant in the diet or the blood lipids, when a diet is rich in saturated fats.
